# Molecular Characterization of Ovarian Yolk Sac Tumor in a Postmenopausal Woman: Implications for Pathogenesis

**DOI:** 10.1111/jog.70422

**Published:** 2026-07-26

**Authors:** Mihiro Dejima, Minoru Nagashima, Mamiko Onuki, Miki Kushima, Akihiko Sekizawa, Koji Matsumoto

**Affiliations:** ^1^ Department of Obstetrics and Gynecology Showa Medical University School of Medicine Tokyo Japan; ^2^ Department of Pathology Koto Toyosu Hospital, Showa Medical University Tokyo Japan

**Keywords:** carcinoma, mutation, ovarian germ cell tumor, ovarian neoplasms, postmenopause

## Abstract

Ovarian yolk sac tumor is a rare malignant germ cell tumor that typically occurs in young women. In postmenopausal women, yolk sac tumors are extremely rare and have a poor prognosis. These tumors may arise from different biological mechanisms, as germ cells are absent in postmenopausal ovaries. We report a 59‐year‐old woman with pure ovarian yolk sac tumor, without epithelial carcinoma components. Immunohistochemistry showed positive SALL4, AFP, and Glypican‐3 and negative PAX8, confirming germ cell tumor differentiation. The patient developed liver metastasis and peritoneal dissemination resistant to platinum‐based chemotherapy. Comprehensive genomic profiling revealed somatic mutations in *ARID1A, CTNNB1*, *NFE2L2*, and *PIK3CA*. These genomic alterations are commonly observed in ovarian clear cell and endometrioid carcinomas, suggesting a somatic, possibly epithelial, origin for postmenopausal yolk sac tumors. These findings suggest that postmenopausal yolk sac tumors may be epithelial carcinomas with yolk sac tumor‐like features, showing genetic and therapeutic similarities to clear cell carcinoma.

## 1 Introduction

Malignant germ cell tumors differ from epithelial ovarian cancers in several respects, including younger age at onset, rapid growth, predominantly unilateral localization, and a generally favorable prognosis [1]. Ovarian yolk sac tumors (YSTs) are a subset of malignant germ cell tumors that are most commonly diagnosed in young women. In postmenopausal women, YSTs are rare, and these cases exhibit a poorer prognosis compared with those in young women [2, 3]. YSTs in postmenopausal women are unlikely to arise from germ cells because germ cells are not observed in ovaries after menopause. Thus, YSTs may arise from different biological pathways in young and postmenopausal women.

YSTs resemble the morphology of the primitive yolk sac and exhibit a variety of histological patterns, including microcystic/reticular, endodermal sinus (festoon), solid, alveolar‐glandular, parietal, papillary, polyvesicular vitelline, hepatoid, and myxomatous patterns [4]. In contrast, most tumors in postmenopausal women with YSTs present with coexisting epithelial components. The most commonly reported epithelial components include endometrioid carcinoma (43%), high‐grade serous carcinoma (22%), clear cell carcinoma (13%), mucinous carcinoma (7%), and neuroendocrine tumor (7%) [5]. Approximately 20% of patients have multiple epithelial components and 31% have endometriosis, endometrioid cysts, or atypical endometriosis. The presence of epithelial components in YSTs in postmenopausal women indicates different molecular pathways compared with germ cell tumors in younger patients [6]. Genetic analysis of YST with epithelial components has shown results similar to those of the coexisting epithelial components. The origin of pure YST without epithelial components remains unclear.

Here, we describe a postmenopausal patient with pure YST who underwent comprehensive genomic profiling (CGP). The origin of the YST was inferred from the CGP results. To the best of our knowledge, this case is the first report of CGP findings in a pure YST in a postmenopausal patient.

## 2 Case Presentation

A 59‐year‐old woman visited our hospital because of a mass in her lower abdomen. She had no familial cancer history and experienced menopause at 40 years old. The patient provided written informed consent to be part of the case report. A magnetic resonance imaging scan revealed a 16 × 12 × 10 cm tumor expanding beyond the umbilicus with both solid and cystic components. Ovarian cancer was suspected. A computed tomography scan revealed no metastasis but detected pulmonary thromboembolism (PE). Consequently, anticoagulant therapy was initiated.

**FIGURE 1:**
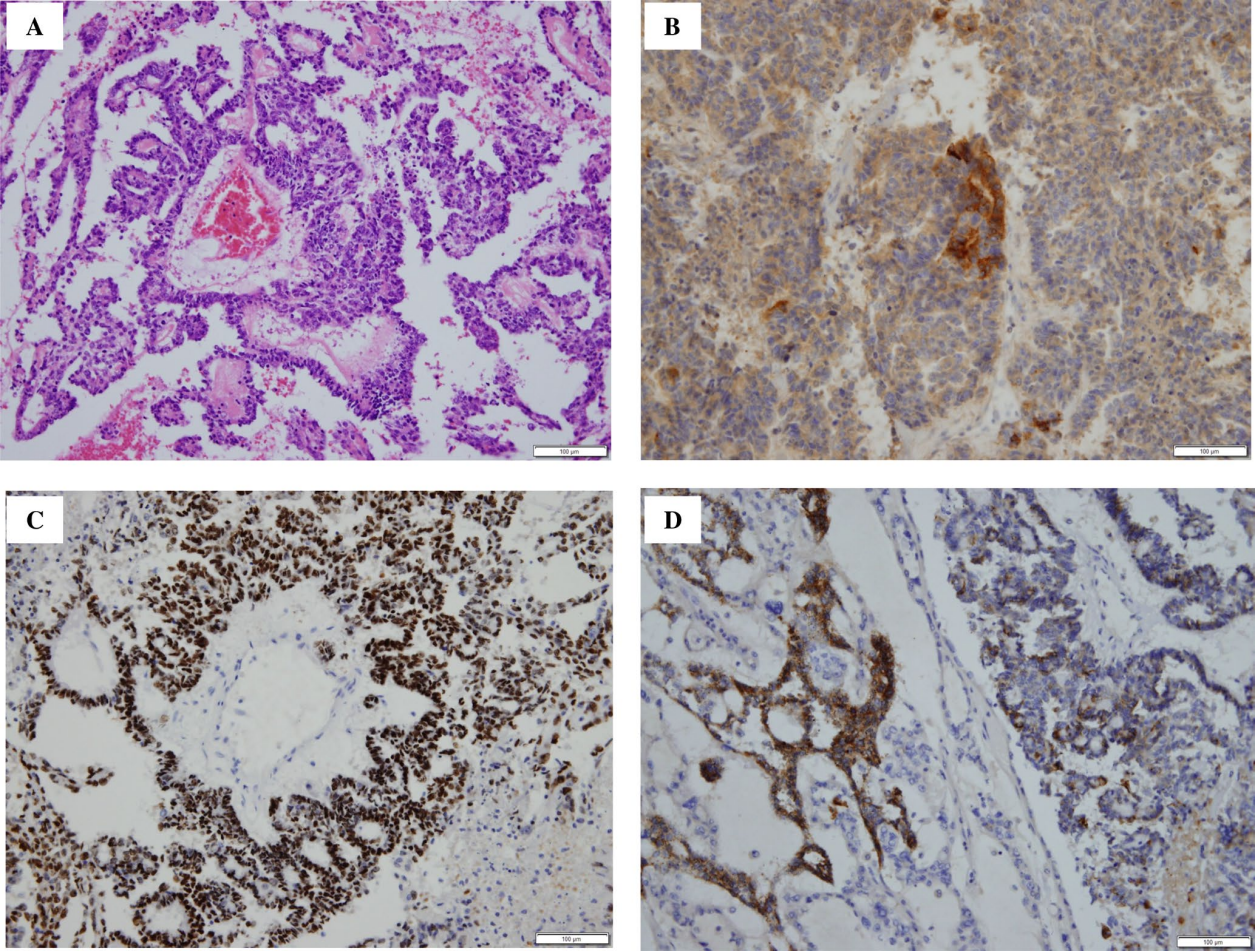
Histopathologic findings. The tumor showed a microcystic and endodermal sinus pattern with Schiller–Duval body (A, H&E, 20×). No epithelial ovarian components were observed. Immunohistochemistry showed positive staining for AFP (B, 20×), SALL4. (C, 20×), and Glypican‐3 (D, 20×).

The first surgery, consisting of a hysterectomy, bilateral salpingo‐oophorectomy, and omentectomy, was performed. Pelvic and para‐aortic lymphadenectomy were not conducted because of the presence of PE. The tumor on the left ovary had ruptured and appeared macroscopically as a solid tumor suggestive of ovarian cancer. Pathological diagnosis confirmed a pure ovarian YST without any epithelial components in the left ovary. We prepared at least one histologic slide per centimeter of tumor size, yielding a total of 18 slides. Immunohistochemistry showed positive AFP, SALL4, and Glypican‐3 and negative PAX8, confirming germ cell tumor differentiation (Figure 1). After improvement of PE, additional staging surgery (pelvic and para‐aortic lymphadenectomy) was performed. A peritoneal dissemination lesion was identified in the peritoneum of the left ovarian vein and resected. Postoperative pathology showed no lymph node metastasis but revealed peritoneal metastasis. The patient was diagnosed with FIGO stage IIIB (pT3bN0M0).

**FIGURE 2:**
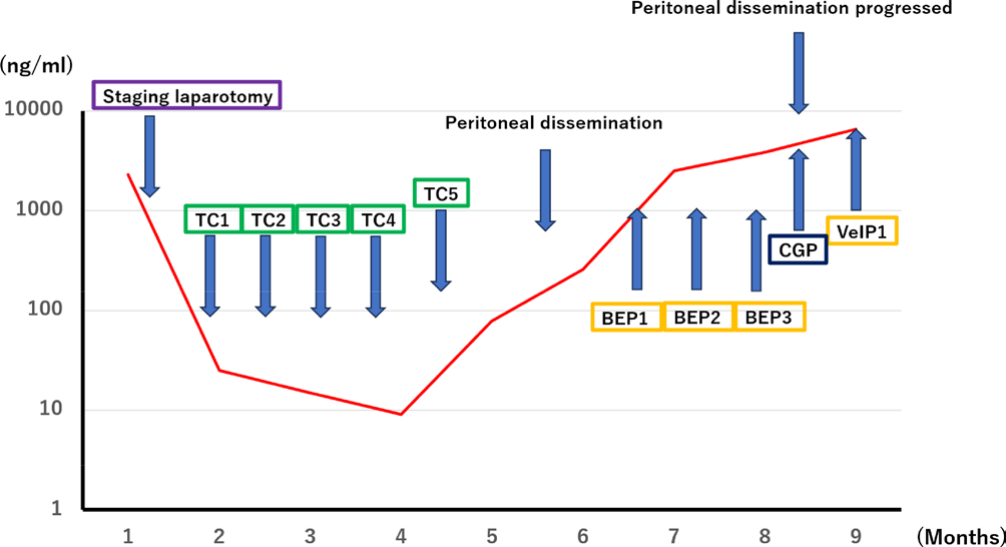
AFP trend following the initial surgery and during treatment. BEP, bleomycin, etoposide, and cisplatin; CGP, comprehensive genomic profiling; TC, paclitaxel and carboplatin; VeIP, vinblastine, ifosfamide, and cisplatin.

After staging laparotomy, TC therapy (paclitaxel 175 mg/m^2^ and carboplatin area under the curve 6, every 3 weeks) was initiated. Serum AFP levels markedly decreased after surgery but began to rise during TC therapy. After five cycles, CT revealed liver metastasis and peritoneal dissemination. The regimen was changed to BEP therapy (bleomycin 30 000 IU on days 2, 9, and 16, etoposide 100 mg/m^2^ on days 1–5, and cisplatin 20 mg/m^2^ on days 1–5, every 3 weeks). However, serum AFP levels continued to rise. CT showed that the peritoneal dissemination worsened after three cycles of BEP. The treatment was changed to VeIP therapy (vinblastine 0.11 mg/kg on days 1 and 2, ifosfamide 1.2 g/m^2^ on days 1–5, and cisplatin 20 mg/m^2^ on days 1–5, every 3 weeks). The treatment was not effective (Figure 2). Because the tumor was resistant to these platinum‐based chemotherapies, the patient underwent CGP to assess responsiveness to molecular targeted therapy. FoundationOne CDx (Chugai Pharmaceutical) was used for CGP. This is a hybrid‐capture–based next‐generation sequencing assay covering the coding exons of 324 cancer‐related genes and selected introns of rearrangement‐prone genes.

The CGP results showed somatic mutations in *ARID1A* c.2632C>T (p.Q878*), *ARID1A* c.4847del (p.A1616Efs*10), *CTNNB1* c.121A>G (p.T41A), *NFE2L2* c.241G>A (p.G81S), *PIK3CA* c.3127A>G (p.M1043V), *PIK3CA* c.3140A>G (p.H1047R) (hotspot). These pathogenic variants are less commonly observed in ovarian malignant germ cell tumors and frequently associated with clear cell carcinoma (*ARID1A* and *PIK3CA*) and endometrioid carcinoma (*ARID1A*, *CTNNB1*, and *PIK3CA*). No druggable variants were identified, and the patient died 13 months after the initial treatment.

## 3 Discussion

In the current patient, the tumor was a pure YST without epithelial components. Somatic variants in *ARID1A*, *CTNNB1*, *NFE2L2*, and *PIK3CA* were identified by CGP. Whole‐exome sequencing analysis of the YST sample revealed several mutated genes, including mutations in *KRAS* and *KIT* and a low frequency of *TP53* mutations [7]. Copy number alterations in driver genes were detected, including deletions in *ARID1A* and *PARK2* and amplifications in *ZNF217*, *CDKN1B*, and *KRAS*. The genetic mutations of germ cell tumors are different from those observed in this case. In ovarian endometrioid carcinoma, which is often associated with postmenopausal YST, the common genetic mutations include mutations in *KRAS*, *PIK3CA*, *PTEN*, *CTNNB1*, *ARID1A*, and *TP53* [8]. The common genetic mutations of ovarian clear cell carcinoma include mutations in *ARID1A*, *PIK3CA, TERTp, KRAS, TP53, POLE*, and *ATM* [9]. In endometriosis, somatic mutations of *KRAS* and *PIK3CA* are commonly observed [10]. While the tumor in the current case lacked epithelial components, the somatic mutations were more similar to those found in endometrioid carcinoma or clear cell carcinoma than those in malignant germ cell tumors. This is supported by the observation that *ARID1A*, *PIK3CA*, and *CTNNB1* alterations are frequently detected in ovarian clear cell and endometrioid carcinomas but are uncommon in malignant germ cell tumors [7, 8, 9]. These findings support the hypothesis that pure YSTs in postmenopausal women may originate from epithelial ovarian cancer. In support of this concept, a recent study found that some pure YSTs in older women harbored diverse genomic alterations, including *DICER1*, *PIK3R1*, *PTPRT*, *PMS1*, *TP53*, *PTEN*, *ARID1A*, *ARID1B*, *FGFR2*, and *CTNNB1* [11]. Hall et al. identified somatic mutations in *CTNNB1*, *PIK3R1*, and *PTEN* in a case of YST with associated endometrioid carcinoma [4]. The authors reported that these mutational patterns are similar to those seen in pure epithelial counterparts, suggesting somatic derivation of the germ cell component. Skala et al. described six cases of YST with epithelial ovarian tumors and endometriosis or lesions suspicious for endometriosis involvement [12]. Somatic mutations were identified in *CTNNB1*, *FBXW7*, *FGFR2*, *RB1*, *PIK3CA*, *PTEN*, and *TP53*. These findings support the notion that the germ cell tumor component of these tumors is often somatically derived. These two cases, like the present case, harbored the *PIK3CA* c.3140A>G (p.H1047R), a hotspot mutation.

BEP is generally considered the standard chemotherapy regimen for ovarian YST, although the number of cycles varies according to residual disease and treatment response [13]. Nevertheless, treatment for YST in postmenopausal women is particularly challenging because of the rarity of these tumors and the lack of established therapeutic protocols. Many patients undergo platinum‐based chemotherapy such as TC or BEP, but the response rate is low and some cases show aggressive progression [2]. Serum AFP levels are elevated in most cases and useful for monitoring treatment response and disease recurrence. Among the 14 previously reported cases of pure YST in postmenopausal patients with known clinical outcomes, 11 experienced recurrence or death, indicating a very poor prognosis (Table 1) [2, 3, 5, 14, 15, 16, 17, 18, 19]. Of the 10 patients who received chemotherapy, 6 were treated with BEP and 2 with cisplatin and etoposide. While pure YST without epithelial components is usually treated by germ cell tumor protocols, the overall prognosis remains poor. For the present case, TC therapy, a standard treatment for epithelial tumors, was chosen as the initial treatment. However, the disease progressed. The regimen was changed to BEP and then VeIP, but these germ cell tumor‐based treatments were completely ineffective. Therefore, treatment based on epithelial tumor protocols may have been worth considering. Larger studies are needed to identify the optimal treatment approach. In addition to their potential role in tumor pathogenesis, these genomic alterations may have therapeutic implications. *ARID1A* deficiency and *PIK3CA* mutations have been associated with potential sensitivity to immune checkpoint inhibitors [20] and PI3K/AKT/mTOR pathway‐targeted therapies in ovarian clear cell carcinoma [21]. Although the clinical utility of these approaches in postmenopausal YST remains unknown, CGP may provide useful information for future therapeutic development in this rare disease.

**TABLE 1:** Summary of the literature on post‐menopausal patients with pure yolk sac tumors.

Case	Author	Year	Age	AFP	Stage	Chemotherapy	Outcome
1	Pliskow [11]	1993	54	n/a	n/a	BEP × 3	NED 24 m
2	Oh [12]	2001	75	17 318	IIIC	EP × 3	DOD 4 m
3	Filiz [13]	2003	76	n/a	II	BEP × 4	DOD 48 m
4	Roth [4]	2011	60	2772	IC	TC × 2	NED 14 m
5	Roma and Przybycin [14]	2014	70	n/a	n/a	n/a	AWD 7 m
6	Parker [15]	2014	60	11 677	III	EP × 2, POMB/ACE	AWD 17 m
7	Boussios [2]	2015	67	31 014	n/a	TC × 1, BEP × 1, TG × 1, CG × 1	DOD 12 m
8	McNamee [16]	2016	50	n/a	IC	n/a	NED 22 m
9	McNamee [16]	2016	40	n/a	IIIB	n/a	DOD 27 m
10	McNamee [16]	2016	42	n/a	IVB	n/a	DOD 8 m
11	Wang [1]	2018	55	14.42	IC	BEP × 6	DOD 30.8 m
12	Wang [1]	2018	60	16 331	IIC	BEP × 4	NED 40.6 m
13	Wang [1]	2018	55	4722	IIC	BEP × 5	DOD 18.5 m
14	Wang [1]	2018	50	n/a	n/a	DDP × 3, FP × 1	DOD 8.5 m

Abbreviations: ACE, actinomycin D, cyclophosphamide, and etoposide; AWD, alive with disease; BEP, bleomycin, etoposide, and cisplatin; CG, carboplatin and gemcitabine; DDP, cisplatin, adriamycin, and 5‐fluorouracil; DOD, dead of disease; EP, cisplatin and etoposide; FP, 5‐fluorouracil and cisplatin; m, months; NED, no evidence of disease; POMB, methotrexate, vincristine, cisplatin, and bleomycin; TC, paclitaxel and carboplatin; TG, paclitaxel and gemcitabine.

These findings suggest that YSTs in postmenopausal women may represent epithelial carcinomas with YST‐like features. Although they are pathologically similar to YSTs in younger patients, their genetic profiles and resistance to platinum‐based chemotherapy indicate a closer resemblance to clear cell carcinoma.

## Author Contributions

**Mihiro Dejima:** conceptualization, writing – original draft, methodology, investigation. **Koji Matsumoto:** formal analysis, supervision. **Mamiko Onuki:** formal analysis, supervision. **Minoru Nagashima:** writing – review and editing, writing – original draft, conceptualization, methodology, investigation. **Miki Kushima:** data curation, investigation. **Akihiko Sekizawa:** formal analysis, supervision.

## Funding

The authors have nothing to report.

## Disclosure

An earlier version of this article was presented at the 77th Annual Congress of the Japan Society of Obstetrics and Gynecology, which was held in Okayama, Japan, on 23–25 May 2025.

## Ethics Statement

This study was approved by the Institutional Review Board of Showa Medical University.

## Consent

Written informed consent for publication was obtained from the patient.

## Conflicts of Interest

The authors declare no conflicts of interest.

## Data Availability

Data sharing not applicable to this article as no datasets were generated or analyzed during the current study.

## References

[1] I. Saani , N. Raj , R. Sood , et al., “Clinical Challenges in the Management of Malignant Ovarian Germ Cell Tumours,” International Journal of Environmental Research and Public Health 20 (2023): 6089. 37372675 10.3390/ijerph20126089 PMC10298722

[2] Y. Wang , J. Yang , M. Yu , et al., “Ovarian Yolk Sac Tumor in Postmenopausal Females: A Case Series and a Literature Review,” Medicine 97 (2018): e11838. 30113473 10.1097/MD.0000000000011838 PMC6112915

[3] S. Boussios , A. Attygalle , S. Hazell , et al., “Malignant Ovarian Germ Cell Tumors in Postmenopausal Patients: The Royal Marsden Experience and Literature Review,” Anticancer Research 35 (2015): 6713–6722. 26637887

[4] A. Cheung , S. Shah , J. Parker , et al., “Non‐Epithelial Ovarian Cancers: How Much Do we Really Know?,” International Journal of Environmental Research and Public Health 19 (2022): 1106.35162125

[5] K. C. Hall , M. D. Post , J. Alldredge , D. L. Aisner , and A. Berning , “Molecular Evidence for Epithelial Origin of Mixed Ovarian Epithelial‐Germ Cell Neoplasms; Report of 2 Cases and Review of Literature,” International Journal of Gynecological Pathology 42 (2023): 403–413. 36305517 10.1097/PGP.0000000000000913 PMC10140189

[6] L. M. Roth , A. Talerman , T. Levy , O. Sukmanov , and B. Czernobilsky , “Ovarian Yolk Sac Tumors in Older Women Arising From Epithelial Ovarian Tumors or With no Detectable Epithelial Component,” International Journal of Gynecological Pathology 30 (2011): 442–451. 21804392 10.1097/PGP.0b013e3182164386

[7] M. T. Pinto , M. G. Erias , A. G. S. Vieira , et al., “Molecular Biology of Pediatric and Adult Ovarian Germ Cell Tumors; a Review,” Cancers 15 (2023): 2990. 37296950 10.3390/cancers15112990 PMC10251860

[8] P. Cybulska , A. D. C. Paula , J. Tseng , et al., “Molecular Profiling and Molecular Classification of Endometrioid Ovarian Carcinomas,” Gynecologic Oncology 154 (2019): 516–523. 31340883 10.1016/j.ygyno.2019.07.012 PMC6736779

[9] Y. Shibuya , H. Tokunaga , S. Saito , et al., “Identification of Somatic Genetic Alterations in Ovarian Clear Cell Carcinoma With Next Generation Sequencing,” Genes, Chromosomes & Cancer 57 (2018): 51–60. 29044863 10.1002/gcc.22507

[10] K. Suda , H. Nakaoka , K. Yoshihara , et al., “Clonal Expansion and Diversification of Cancer‐Associated Mutations in Endometriosis and Normal Endometrium,” Cell Reports 24 (2018): 1777–1789. 30110635 10.1016/j.celrep.2018.07.037

[11] T. A. Numan , B. M. Ronnett , L. Haley , et al., “Clinicopathologic and Molecular Analysis of Malignant Neoplasms With Yolk Sac Tumor Differentiation in Women 40 Years of Age and Older,” American Journal of Surgical Pathology 49 (2025): 686–700. 40202333 10.1097/PAS.0000000000002389

[12] S. L. Skala , C. J. Liu , A. M. Udager , and A. P. Sciallis , “Molecular Characterization of Uterine and Ovarian Tumors With Mixed Epithelial and Germ Cell Features Confirms Frequent Somatic Derivation,” Modern Pathology 33 (2020): 1989–2000. 32404953 10.1038/s41379-020-0548-6

[13] S. Boussios , G. Zarkavelis , E. Seraj , I. Zerdes , K. Tatsi , and G. Pentheroudakis , “Non‐Epithelial Ovarian Cancer: Elucidating Uncommon Gynaecological Malignancies,” Anticancer Research 36 (2016): 5031–5042. 27798862 10.21873/anticanres.11072

[14] S. Pliskow , “Endodermal Sinus Tumor of the Ovary: Review of 10 Cases,” Southern Medical Journal 86 (1993): 187–189. 7679524 10.1097/00007611-199302000-00009

[15] C. Oh , A. Kendler , and E. Hernandez , “Ovarian Endodermal Sinus Tumor in a Postmenopausal Woman,” Gynecologic Oncology 82 (2001): 392–394. 11531302 10.1006/gyno.2001.6286

[16] G. Filiz , S. Ozuysal , and T. Bilgin , “Ovarian Endodermal Sinus Tumor in a 76‐Year‐Old Woman,” Journal of Obstetrics and Gynaecology Research 29 (2003): 309–311. 14641700 10.1046/j.1341-8076.2003.00120.x

[17] A. A. Roma and C. G. Przybycin , “Yolk Sac Tumor in Postmenopausal Patients: Pure or Associated With Adenocarcinoma, a Rare Phenomenon,” International Journal of Gynecological Pathology 33 (2014): 477–482. 25083963 10.1097/PGP.0000000000000078

[18] V. L. Parker , P. Sanderson , V. Naik , C. Quincey , and K. Farag , “Post‐Menopausal Presentation of Yolk Sac Germ Cell Tumour,” Gynecologic Oncology Reports 11 (2014): 16–19. 26076087 10.1016/j.gore.2014.11.001 PMC4434160

[19] T. McNamee , S. Damato , and W. G. McCluggage , “Yolk Sac Tumours of the Female Genital Tract in Older Adults Derive Commonly From Somatic Epithelial Neoplasms: Somatically Derived Yolk Sac Tumours,” Histopathology 69 (2016): 739–751. 27334714 10.1111/his.13021

[20] T. Fukumoto , N. Fatkhutdinov , J. A. Zundell , et al., “HDAC6 Inhibition Synergizes With Anti‐PD‐L1 Therapy in ARID1A‐Inactivated Ovarian Cancer,” Cancer Research 79 (2019): 5482–5489. 31311810 10.1158/0008-5472.CAN-19-1302 PMC6825538

[21] K. Zheng , G. Jin , R. Cao , et al., “Targeting on the PI3K/mTOR: A Potential Treatment Strategy for Clear Cell Ovarian Carcinoma,” Cancer Chemotherapy and Pharmacology 95 (2025): 21. 39792198 10.1007/s00280-024-04748-3 PMC11723846

